# Enhancing sleep, wakefulness, and cognition with transcranial photobiomodulation: a systematic review

**DOI:** 10.3389/fnbeh.2025.1542462

**Published:** 2025-07-31

**Authors:** Naomi L. Gaggi, Zamfira Parincu, Anna Peterson, Courtney O’Brien, Korey Kam, Umit Tural, Indu Ayappa, Andrew W. Varga, Dan V. Iosifescu, Ricardo S. Osorio

**Affiliations:** ^1^Grossman School of Medicine, New York University, New York, NY, United States; ^2^Icahn School of Medicine at Mount Sinai, New York, NY, United States; ^3^Nathan S. Kline Institute for Psychiatric Research, Orangeburg, NY, United States

**Keywords:** transcranial photobiomodulation, sleep, wakefulness, cognition, sleep disorders

## Abstract

Disruptions in sleep are common across clinical populations, particularly those with neurological and psychiatric disorders, making restorative sleep and sustained wakefulness a public health priority. Sleep is essential for brain function, impacting cognition in addition to serving as a critical factor in memory consolidation and healthy aging. Neuromodulation via transcranial photobiomodulation (t-PBM) increases cerebral mitochondrial activity and blood flow. These effects may underlie improvements in sleep quality and wakefulness observed after t-PBM. In this systematic review, we summarize the current literature across clinical and healthy populations, which describes t-PBM’s potential to improve sleep, wakefulness, and cognition. The scope of this review also includes t-PBM’s effect on the brain’s glymphatic system and blood flow, the potential of this strategy to augment alertness, wakefulness, and associated cognitive processes, and the suggestion for targeted t-PBM application for future research based on the underlying neurobiological mechanisms of t-PBM and wakefulness across diverse clinical populations.

## 1 Introduction

Disruptions in sleep and wakefulness are prevalent in clinical populations (Pérez-Carbonell et al., 2022), particularly those with neurological and psychiatric disorders. These disruptions affect many aspects of daily life, including memory consolidation and healthy aging (Hamel et al., 2023; Mullins et al., 2021). The relationship between unhealthy sleep and wakefulness with associated cognitive functions (e.g., memory, executive function) has become a concern in sleep-related disorders. For instance, disruptions have increasingly been recognized as a potential risk factor for future cognitive decline and dementia (Osorio et al., 2015), making the enhancement of wakefulness both a public health priority and a clinically relevant concern for many individuals. One emerging neuromodulation technique, transcranial photobiomodulation (t-PBM), has shown promising neurobiological effects in many populations (Dole et al., 2023; Hamblin, 2016). t-PBM boosts cerebral mitochondrial function, increases adenosine triphosphate (ATP) production, and enhances cerebral blood flow (Hamblin, 2016), which are all mechanisms that may underpin observed improvements in wakefulness, sleep quality, and associated cognition across diverse populations.

The underlying principle behind t-PBM is based on the effects of light on cellular metabolism (Hamblin, 2016). In brief, t-PBM activates light-sensitive ion channels, which are absorbed by cytochrome c oxidase (CCO), the terminal enzyme in the mitochondrial electron transport chain. Absorption of photons by CCO increases energy production and boosts brain metabolism. Numerous tissue-culture and animal studies have shown that t-PBM upregulates CCO activity (Chung et al., 2012; Huang et al., 2009; Karu, 2000; Karu et al., 2005; Wong-Riley et al., 2005), increases nitric oxide production (Chung et al., 2012; Leung et al., 2002), and accelerates cortical ATP production (Karu, 2000; Wong-Riley et al., 2005). These effects trigger secondary neuronal and tissue-specific processes, including enhanced cerebral blood flow and oxygenation.

Nitric oxide release, in particular, plays a critical role in vasodilation through the opening of calcium-activated potassium channels (Hamblin, 2008; Hennessy and Hamblin, 2017), which reduces endothelial calcium concentrations and promotes vasodilation with little-to-no side effects. t-PBM has also been shown to increase regional cerebral blood flow and oxygenation in healthy adults (Tian et al., 2016; Hamblin, 2018). Dole et al. (2023) recently summarized the cerebral hemodynamic changes following t-PBM, highlighting changes in brain activity and cerebral blood flow. Moreover, individuals from both clinical (i.e., all groups who have clinical diagnoses) and healthy populations who underwent t-PBM treatment demonstrated improvements in cognitive performance, including reaction time, accuracy, and overall task performance (Lee et al., 2023; Salehpour et al., 2022). These findings suggest that t-PBM may enhance wakefulness and alertness, likely owed to its positive effects on cerebral circulation and metabolism. Although more research is needed to understand t-PBM’s neurobiological effect on enhancing wakefulness and alertness in humans, preclinical animal models have provided foundational insights into the underlying mechanisms.

In preclinical animal models, t-PBM has demonstrated potential for improving both sleep quality and brain function (Valverde et al., 2023; Moro et al., 2022). During wakeful states, t-PBM has been applied to various mouse models and has demonstrated several effects supporting t-PBM’s potential role in improving sleep and alertness, including better performance on behavioral tasks and increased glymphatic drainage and toxin clearance (Salehpour et al., 2022; Semyachkina-Glushkovskaya et al., 2021a; Mohammed and Khadrawy, 2022; Hennessy and Hamblin, 2017). Interestingly, recent studies suggest that t-PBM may have different effects during sleep compared to wakefulness in rodent models, possibly improving the clearance of fluid-filled waste products and debris from the brain into the glymphatic system (Semyachkina-Glushkovskaya et al., 2021a). For example, in a preclinical animal model of Alzheimer’s disease (AD), t-PBM reduced β-amyloid accumulation and cognitive decline during sleep, with less pronounced effects during the day versus the nighttime (Semyachkina-Glushkovskaya et al., 2021b), suggesting that time of day may have a different neurophysiological effect. These findings have been supported by other studies demonstrating increased clearance of cerebrospinal fluid substances following t-PBM treatment (Semyachkina-Glushkovskaya et al., 2020; Zinchenko et al., 2019).

However, not all studies have reported a positive outcome. In a recent randomized, blinded study, using t-PBM during wakefulness states in a preclinical mouse model of AD (5 × FAD), no differences were found in behavioral tests between the 5 × FAD mice treated with t-PBM and the control group, as well as no difference in histological tests on amyloid load, neuronal loss, or microglial response (Sipion et al., 2023). Importantly, the application of t-PBM during sleep was not performed, which leaves the arousal-dependent effects of t-PBM still underexplored. The discrepancy in the efficacy of t-PBM in preclinical animal models may be clarified by focusing on t-PBM application during sleep and standardizing treatment parameters. Subsequent studies of t-PBM during sleep should also assess changes to objective measures of post-intervention sleep such as duration, sleep stage and sleep oscillatory patterns.

Preclinical animal research has the potential to explore t-PBM application during both sleep and wakeful states to inform on the neurobiological mechanisms of t-PBM and associated cognitive consequences. Chronic sleep disruption and short sleep can lead to poor attention, cognition and memory impairment (Van Someren et al., 2015). Recently, an experimentally induced animal model of chronic sleep disruption found reduced glymphatic clearance and vasomotion from the cortex and subcortical regions but not the hippocampus (Deng et al., 2024). Interestingly, impaired glymphatic clearance was detected after 30 days of chronic sleep disruption but not after 3 days of sleep disruption suggesting glymphatic compensation in the short term. If individuals are deprived of quality sleep, and the brain does not clear its waste effectively (Yan et al., 2021), this can lead to a lack of attention, slower cognitive function, compromised memory recall and consolidation, and impaired motor functions (Scott et al., 2006; Varga et al., 2014a; Voumvourakis et al., 2023). More research is needed to understand the role of glymphatic clearance in the neurobiological mechanism of t-PBM and how this may contribute to cognitive and sleep-related changes post-treatment. Clinical research including measures of glymphatic clearance using neuroimaging after t-PBM would greatly inform the field and point toward its potential clinical significance.

In both clinical and healthy populations, cerebral blood flow and brain activity changes have been very well-documented (Hamblin, 2016; Dole et al., 2023). Increasing cerebral blood flow and metabolism may represent a non-pharmacological and non-invasive therapy to improve wakefulness and cognition in many clinical groups who may have decreased blood flow and metabolism after disrupted sleep (Wu et al., 2006). Although a few studies have explored the mechanism and quantitative measures of sleep in clinical populations, there is emerging evidence detailed in the current systematic review of potential therapeutic effects of t-PBM on sleep and wakefulness in humans that may arise from several mechanisms. One mechanism is that t-PBM helps to reduce oxidative damage and inflammation (Hamblin, 2016; Cassano et al., 2016), both of which are implicated in various neurological disorders, including those that affect sleep regulation (Atrooz and Salim, 2020). This reduction in oxidative stress could play a significant role in improving both sleep quality and cognitive function, particularly in clinical populations. Though this has not been directly tested, cerebral blood flow and brain activity may potentially underlie the reported increased cognition and wakefulness after t-PBM. However, randomized, controlled trials and inclusion of neuroimaging measures neuroimaging and objective physiological measures are needed in future research to understand this relationship.

Another potential, yet speculatory mechanism of t-PBM’s effect on sleep may involve t-PBM’s effects on neural circuits responsible for regulating sleep-wake cycles and cognitive processing during the day. By increasing cerebral blood flow and enhancing hemodynamic responses in frontal regions of the brain (Dmochowski et al., 2020; Gaggi et al., 2024) that are interconnected with cognitive and sleep-wake circuits (Scammell et al., 2017), t-PBM may optimize brain function and blood flow regulation during wakefulness. Increasing cerebral blood flow is the most well-studied effect of t-PBM in both humans and animals (Hamblin, 2016), and evidence suggests that improving cerebral blood flow during the day can promote wakefulness and alertness. t-PBM promotes ATP production, which in turn increases cerebral blood flow (Hamblin, 2016). Enhanced blood flow supports neural activity (Iadecola, 2017) and this blood flow and metabolism relationship is impaired after sleep deprivation (Rab-Bábel et al., 2025). Increasing cerebral blood flow may promote wakefulness and alleviate fatigue and cognitive sluggishness.

One of the most common targets of t-PBM, especially when using near-infrared low-level laser light, is the frontal cortex (Dole et al., 2023) due to its feasibility (i.e., no hair, suitable skull thickness) and being a key component in many cognitive processes (Buckner et al., 1999; Stuss and Benson, 2019). Research on insomnia has shown decreased cerebral blood flow and metabolic function in regions of the brain involved in memory, executive function, and alertness, such as the frontal lobes (Zhou et al., 2019; Wu et al., 2006; Thomas et al., 2000). Specifically, Wu et al. (2006) demonstrated reductions in both glucose metabolism and cerebral blood flow in the frontal regions of the brain after sleep deprivation, with only partial recovery after a single night of recovery sleep. This is supported by other work implicating the frontal lobe as heavily influenced by slow-wave sleep (SWS) and a key factor in maintaining functioning during wakefulness and cognitive function (Dijk, 2009; Thompson-Schill et al., 2009). Together, these results emphasize the critical role of the frontal lobe in both sleep, wakefulness, and associated cognitive processes, suggesting that improving cerebral blood flow via t-PBM could alleviate the cognitive deficits associated with sleep deprivation and disorders such as insomnia.

Transcranial photobiomodulation may also improve sleep quality by reducing sleep disruptions and increasing the duration of SWS and rapid eye movement (REM) sleep, both of which are essential for memory consolidation, cognitive processing, and toxin clearance (Ahuja et al., 2018; Walker, 2009; Xie et al., 2013; Varga et al., 2014b; Mullins et al., 2025; Carvalho et al., 2024). By optimizing sleep quality, t-PBM can also sustain wakefulness, which may make it easier to maintain alertness during the day. This is especially relevant for conditions such as chronic fatigue, excessive daytime sleepiness, and cognitive impairments associated with sleep deprivation. t-PBM may also help to balance sleep-promoting (e.g., GABAergic) and wake-promoting (e.g., glutamatergic) neurotransmitters throughout the day, which could further optimize the sleep-wake cycle. While there is some evidence that t-PBM may modulate GABAergic (e.g., Zomorrodi et al., 2019) and glutamatergic (e.g., Zhang et al., 2021) neurotransmitters, direct evidence is needed to support the hypothesis that this mechanism is related to sleep, wakefulness, and/or cognition.

Transcranial photobiomodulation is sometimes combined with intranasal photobiomodulation (i-PBM); using i-PBM, subcortical structures may be able to be targeted. Whether through t-PBM’s downstream effects on subcortical structures or i-PBM’s potential to reach subcortical regions, red light has been shown to increase melatonin secretion in the pineal gland (Yeager et al., 2007), which may help to regulate the internal circadian clock and improve sleep by increasing melatonin release. Chronic fatigue and cognitive sluggishness are often linked to neuroinflammation and oxidative stress (e.g., Morris and Maes, 2014; Lee et al., 2018; Irwin, 2019; Irwin and Vitiello, 2019; Carvalho et al., 2022; Fernandes et al., 2024; Veasey et al., 2004), which impair brain function and alertness, and both are improved after t-PBM applications. Sleep abnormalities are a common complaint in patients experiencing chronic fatigue/myalgia with patients having poor sleep efficiency and decreased Stage 2 sleep, more Stage 3, and longer REM sleep latency among other differences (Jackson and Bruck, 2012; Mohamed et al., 2023). t-PBM’s antioxidant and anti-inflammatory effects, particularly its influence on the glymphatic system (Salehpour et al., 2022; van Hattem et al., 2025), may help mitigate these impairments by reducing reactive oxygen species (Lin et al., 2024). By decreasing neuroinflammation and oxidative damage, t-PBM may contribute to better quality sleep, improved mental clarity, and enhanced sustained wakefulness. However, more evidence is needed to support these claims and hypotheses, as t-PBM is recently emerging as a novel neuromodulation strategy in sleep research.

Cognitive health is heavily influenced by sleep and wakefulness, including memory consolidation, memory encoding, and cognitive reserve (Diekelmann and Born, 2010; Stickgold, 2005; Balsamo et al., 2024). Restorative sleep, including both SWS and REM, is critical for memory processing. Diekelmann and Born (2010) review many hypotheses, all of which highlight that SWS and REM may complement each other to consolidate memories and ensure restorative sleep. One hypothesis includes the critical role of SWS, which is characterized by slow wave oscillations and synchronized neuronal activity. The hypothesis suggests that SWS may place a role in active system consolidation and during this process new information can be integrated with long-term memories leading to changes in the neural representations. Recently, Mullins et al. (2025) demonstrated that individuals with obstructive sleep apnea who had increased regional slow oscillations during continuous positive airway pressure treatment showed improvements in overnight memory during stable SWS. This suggests that slow oscillations may be critical for overnight memory processing. Further, sleep disruptions that reduce slow oscillations may yield change in spatial navigation performance and memory. Restorative sleep is also critical for post-arousal cognitive processes; disrupted sleep has been shown to affect both short-term and long-term cognitive processes during wakefulness, including attention, vigilance, and executive function (Wickens et al., 2015; Medic et al., 2017). There has also been evidence that sleep disordered breathing, and disrupted sleep may affect the age of onset for cognitive decline and may serve as a risk factor for AD (Osorio et al., 2015; Macedo et al., 2017).

In summary, the emerging evidence supporting t-PBM as a therapeutic tool for improving both sleep quality and wakefulness is promising. However, the underlying mechanisms are speculative as they are still in their infancy and more research is needed to establish t-PBM as a tool for sleep and wakefulness processes. By enhancing cerebral blood flow, increasing mitochondrial function, and reducing oxidative stress, t-PBM may provide a safe, non-pharmacological and non-invasive means of addressing sleep disorders and related cognitive dysfunctions that could be applied at home. While most research has focused on its effects during wakefulness and most of the literature is based on animal models, there is growing support for its potential to improve sleep quality and wakefulness in clinical populations with comorbid sleep disruptions. Further studies are needed to explore these mechanisms in greater depth and to assess the long-term effects of t-PBM on both sleep and cognitive function. To date, 17 studies have been conducted that explored sleep quality as an outcome measure after t-PBM, including individuals with traumatic brain injury (Naeser et al., 2011; Morries et al., 2015; Naeser et al., 2014; Martin et al., 2021; Naeser et al., 2023; Carneiro et al., 2019; Chao, 2019), cognitive decline (Nizamutdinov et al., 2021; Saltmarche et al., 2017; Zhao et al., 2022), mood disorders (i.e., depression or anxiety; Maiello et al., 2019; Hao et al., 2024; Guu et al., 2025), healthy athletes (Zhao et al., 2012), bipolar disorder (Mannu et al., 2019), Parkinson’s disease (Liebert et al., 2024), and children with autism spectrum disorder (Pallanti et al., 2022). In this review, we aim to detail findings across clinical and healthy populations. The purpose of summarizing sleep-related findings across populations after t-PBM by this systematic review is to 1) highlight the importance of the promise of t-PBM’s improvement of sleep, alertness, and cognition and 2) emphasize the importance of future studies to understand the neurobiology of t-PBM’s effect on sleep and wakefulness.

## 2 Methods

This systematic review aims to summarize the current literature on sleep, wakefulness, and associated cognitive changes across both clinical and healthy populations. All relevant literature including human populations to date was considered for inclusion in this review. Given the heterogeneity of the existing studies–ranging widely in terms of populations, diagnoses, methodologies, and outcome measures–we organized the literature into three thematic subgroups (see below for more detail). This approach allowed for a more structured synthesis and facilitated the identification of common patterns and gaps within and across study types.

The following steps were taken to identify all relevant literature that included a sleep outcome measure and associated cognitive assessment (please refer to Supplementary Figure 1 for PRISMA flow chart). Studies included in this systematic review were required to meet the following eligibility criteria: (1) PubMed search criteria terms, “photobiomodulation” AND “sleep”; “photobiomodulation” AND “wake,” (2) only human studies (special filter for humans was selected on PubMed), and (3) articles that have full articles available (not abstracts or conference posters). A manual search for *transcranial* photobiomodulation was conducted through all articles and review articles were excluded. Articles were included in this review by using PubMed as the initial search engine; Scopus, Google Scholar, or referenced studies within identified literature were utilized to collect the remaining articles that fit the search criteria from 1987 to March 2025. Out of the 31 results from the “photobiomodulation” AND “sleep,” “photobiomodulation” AND “wake” searches, one was excluded because it was a protocol report; 11 were excluded because they were review articles and unrelated to the purpose of this review; and 11 were excluded because the application of photobiomodulation was not transcranial. From this search, eight studies were included as eligible for inclusion in the current review. 11 additional studies were included in the list of studies eligible for inclusion in the current review after searches in Google Scholar and Scopus using the same terms as above. If any eligible articles were referenced within the included studies but not retrieved in the original search, efforts were made to locate and include those as well. Out of 19 eligible results, 17 were included in this review; two conference abstracts were excluded (Bogdanova et al., 2017; Liebel et al., 2022). These conference abstracts are discussed in the “Section 4 Discussion” due to the relevance of this review. Refer to Supplementary Figure 1 and Supplementary Table 1 for detailed information on the included literature. Tables 1–3 correspond to the sections below and include details on study parameters.

**TABLE 1:** Effects of transcranial photobiomodulation on sleep and wakefulness in traumatic brain injury.

References	Type of TBI	Study design	Number of treatment	Subjects enrolled	Sex	Age	NIR device (LED/laser)	PW/CW
Naeser et al., 2011	Chronic TBI (closed-head)	Case report	Participant 1: approximately 31 in clinic (then at home-treatment daily for 5 years). Participant 2: 28.	2	100% female	Mean age: 55.5 years SD: 4.95	LED (2 devices used)	CW
Naeser et al., 2014	Chronic mild TBI	Open-protocol pilot; case series	3/week for 6 weeks	11	45% female	Mean age: 44.3 years SD: 13.7	2 LED console units	CW
Naeser et al., 2023	Possible CTE	Open-protocol pilot	3 times a week for 6 weeks	4	100% male	Mean age 62.7 years	LED (t-PBM and iPBM)	Protocol A: CW. Protocol B: PW; 40 Hz. Protocol C: CW.
Morries et al., 2015	Chronic mild to moderate TBI	Retrospective case series	8–12 min., 10 visits (6/10 ptps), 20 visits (4/10 ptps)	10	50% female	N/A	Laser (2 devices used)	CW or PW
Carneiro et al., 2019	Severe TBI	Open-protocol	3 times a week for 6 weeks	10	10% female	Mean age 37.8	LED	CW
Chao, 2019	Gulf war illness	Open-protocol pilot	Every other day for 12 weeks	2	100% male	Mean age 61	LED, vielight neuro alpha	PW, 10 Hz, 50% duty cycle
Martin et al., 2021	Gulf war illness	Randomized, sham-controlled, double-blind	2 times a week for 7.5 weeks	48	17% female	Mean age 52 years	3 LED devices (helmet, ear, intranasal)	CW and PW (intranasal) 10 Hz, 50% duty cycle
870/663 nm	Varied	500 mW	Pt. 1: gradually increased from 8 J/cm^2^ to 20 J/cm^2^ Pt. 2: Gradually increased from 9 J/cm^2^ to 10.6 J/cm^2^ to 12 J/cm^2^ to 13.3 J/cm^2^	Bilaterally and over midline sagittal areas.	Subjective	Pt. 1: improved sleep	Pt. 1: time spent on computer Pt. 2: stroop test; Wechsler memory scale- revised	Pt. 1: increased time spent on computer Pt. 2: improvements in executive function, inhibition, and memory
850 nm or 870 nm	20 min	500 mW	13 J/cm^2^	DMN, SN, CEN	Self-report	Improved sleep	Stroop test, California verbal learning test-II, Delis–Kaplan executive function, controlled oral word association test, digit span, forward and backward, Wechsler adult intelligence scale	Improvements in cognitive performance (inhibition, inhibition switching, verbal learning, and memory)
Protocol A: 2 sets of 6 LED cluster heads (9, 633 nm diodes and 52, 870 nm diodes in each cluster head). Protocol B: 4, 810 nm single t-PBM diodes with 1, 810 nm intranasal, and all pulsed at 40 Hz; 1 single diode, 633 nm, and continuous. Protocol C: each LED cluster head: 34, 660 nm, and 35, 850 nm diodes; 5 LED cluster heads on midline, and 5 LED cluster heads on each side.	22–40 min	–	26 J/cm^2^	DMN, SN, CEN	PSQI	Increased SN functional connectivity after 1-month treatment and sleep	Color-word interference (stroop), California verbal learning test-II, trail-making test, controlled oral word association test, Connors’ continuous performance test, brief visuospatial memory test- revised	Improvements in cognition were seen across multiple domains, including verbal learning and memory
810 nm and 980 nm	8–10 min per area; 2–3 areas per subject.	N/A	Ranged from 55 J/cm^2^ to 81 J/cm^2^	Frontal and temporal region	Subjective	Resolved primary and middle insomnia in all participant	–	Examination of the patients that cognition may have improved
630 nm	30 min	830 mW	–	Whole head	Subjective	Insomnia reduction	–	–
810 nm	20 min	100 mW/cm^2^ (posterior), 75 mW/cm^2^ (anterior), 25 mW/cm^2^ (intranasal)	–	–	Insomnia severity Index	6-point and 12-point reduction in ISI		
830 nm (helmet); 633 or 810 nm (intranasal); 870 nm (ear)	30 min	626 (helmet), 90 (ear), 8 and 14.2 (intranasal)	26 (helmet), 2 (Ear), 12 and 10.65 (intranasal)	–	PSQI, Karolinska sleepiness scale, Epworth sleepiness scale, multi-dimensional fatigue inventory	Not significant	Digit span, Delis-Kaplan executive function test trails, color-word interference (stroop), California verbal learning test II, Connor’s continuous performance test II, Rey osterrieth complex figure test	Cognitive improvements post-treatment showed a trend toward significance

TBI, traumatic brain injury; DMN, default mode network; SN, salience network; CEN, central executive network; PSQL, Pittsburgh Sleep Quality Index; CW, continuous wave; PW, pulsed wave; CTE, chronic traumatic encephalopathy; LED, light emitting diodes.

**TABLE 2:** Effects of transcranial photobiomodulation on sleep and wakefulness in cognitive decline.

References	Type of dementia	N	Age	Sex	Study design	Number of treatments	NIR device (LED/laser)	PW/CW
Nizamutdinov et al., 2021	Early and moderate dementia	60	Mean age 74.2 years	40% females	Randomized, sham-controlled, double-blind	2 times a day, daily for 8 weeks	LED	–
Saltmarche et al., 2017	Mild to moderate dementia or AD	5	Mean age 77.6 years	20% female	open-study; at-home and in-clinic	2 times a week for first 2 weeks, 1 times a week for last 10 weeks (transcranial); 1 time a day for 12 weeks (intranasal)	LED	PW, 10 Hz, 50% duty cycle
Zhao et al., 2012	Subjective cognitive decline	58	Mean age 63.95 years	60% female	Randomized, sham-controlled, double-blind	1 time a day for 6 continuous days	Laser	CW
1060–1080 nm	–	6 min	23.1 mW/cm^2^	–	Caregiver daily logs and feedback notes	Sleep duration increased 1 h on average after 8 ± 2 days of the treatment	Logical memory, auditory verbal learning, trail making A and B, Boston naming test, and mini mental status examination	Logical memory, auditory verbal learning, trail making A and B, Boston naming test, and mini mental status examination improved
810 nm	24.6 (transcranial) 13.8 (intranasal) J/cm^2^	20 min	41 (transcranial) 23 (intranasal) mW/cm^2^	Mesial prefrontal cortex, precuneus, posterior cingulate cortex, inferior parietal lobe, and hippocampus.	Daily home treatment journal, self-report and caregiver report	Improved sleep	Mini mental status examination; cognitive behavior scale from the Alzheimer’s disease assessment scale	Improvement on both cognitive tests after treatment
1064 nm	–	12 min	250 mW/cm^2^	Prefrontal cortex	Sleep monitoring with wearable device (sleepart)	Sleep efficiency and the percentage of REM sleep on the fifth day were improved	N-back task	Improvements in reaction time and accuracy

REM, rapid eye movement; CW, continuous wave; PW, pulsed wave; AD, Alzheimer’s disease; LED, light emitting diodes.

**TABLE 3:** Effects of transcranial photobiomodulation on sleep and wakefulness in other populations.

References	Population	Study design	N	Sex	Age	Number of treatments	NIR device (LED/laser)	PW/CW
Zhao et al., 2012	Healthy athletes	Randomized, sham-controlled	20	100% female	Mean age 18.6 years	14 days	LED	CW
Pallanti et al., 2022	Children with autism spectrum disorder	Open-label, at-home	21	38% female	Mean age 9.1 years	5/week for 6 months	LED	PW, 40 Hz
Maiello et al., 2019	Generalized anxiety disorder	Open-label, at-home	15	67% female	Mean age 30 years	1/daily for 8 weeks	LED	CW
Mannu et al., 2019	Bipolar disorder, type I	Open-label	4	50% female	Mean age 38.5 years	2/week for 8 weeks	LED	CW
Liebert et al., 2024	Parkinson’s disease	Waitlist design	7	71% female	Mean age 65.8 years	3/week for 5 years (at-home)	LED	PW
658 nm	30 min	–	Whole body (including head)	30 J/cm^2^	PSQI (Chinese version)	Improvements in global PSQI, subjective sleep quality; group for sleep duration and sleep latency	–	–
810 nm	20 min	–	Medial prefrontal cortex, precuneus area, left and right angular gyrus, ventromedial PFC	65 J/cm^2^ (posterior LED) and 45 J/cm^2^ (anterior LED)	PSQI	Increase in sleep quality post-treatment (*p* < 0.01)	Scala per i disturbi di attenzione/ iperattività per genitori (inattention subscale and hyperactivity/ impulsivity) completed by parents; Montefiore Einstein rigidity scale–revised	Improvements in sleep quality, cognitive and behavioral rigidity, and attention
830 nm	20 min	30 mW/cm^2^	–	36 J/cm^2^	PSQI	Improved sleep latency post-treatment (*p* < 0.02)	–	–
830 nm	20 min	33.2 mW/cm^2^	Dorsolateral PFC	40 J/cm^2^	Self-report	Improved sleep	Self-report	Decreased impulsivity
810 nm	35 min	100 mW/cm^2^ (posterior), 75 mR/cm^2^ (anterior), 25 mW/cm^2^ (nasal)	Posterior, anterior, nasal	–	PDSS	Sleep quality was improved in four out of six participants	Montreal cognitive assessment (MoCA)	All participants had a higher MoCA score at 5 years than at baseline

CW, continuous wave; PW, pulsed wave; LED, light emitting diodes; PFC, prefrontal cortex; PSQL, Pittsburgh Sleep Quality Index; PDSS, Parkinson’s Disease Sleep Scale.

## 3 Results

To synthesize the current literature on sleep, wakefulness, and associated cognitive changes in clinical and healthy populations, the authors categorized the literature into three subgroups (described in “Section 2 Methods”). 17 articles focusing on sleep and wakefulness after t-PBM resulted from our search and studies were categorized into three subgroups: (1) traumatic brain injury, (2) cognitive decline, and (3) other populations.

### 3.1 Traumatic brain injury

Exploration of the effects of t-PBM on populations with traumatic brain injuries (TBI) has produced some evidence of cognitive improvement as well as elicited both subjective and quantifiable reports of improved sleep quality (see Table 1 for study details). Out of seven total studies, three used validated self-report measures of sleep (Naeser et al., 2023; Chao, 2019; Martin et al., 2021), while the rest used subjective assessments (Naeser et al., 2011; Naeser et al., 2014; Morries et al., 2015; Carneiro et al., 2019). In addition, quantitative physiological changes were only recorded in two study using neuroimaging (Carneiro et al., 2019; Naeser et al., 2023) and only one study was a randomized controlled trial (Martin et al., 2021).

Naeser et al. (2011) presented two case reports investigating the cognitive benefits of red and near-infrared t-PBM, applied to the forehead and throughout scalp areas of chronic TBI patients. Both patients demonstrated improvements in mood and cognition as well as subjectively improved sleep quality. Patient One reported improvements in excessive daytime sleepiness and as well as improvement in alertness and was able to focus on computer tasks for up to 40 min after 1 week of treatment. The patient noted a preference for conducting their treatments prior to sleep due to self-reported improvements in sleep quality. After 6 years of treatment, the patient has shown marked improvements in alertness and is able to concentrate on computer tasks for up to 3 h. Patient Two demonstrated cognitive function and attention deficits post-injury, which improved to a level where the patient could return to work full-time after 4 months of treatment. Treatments initially were conducted during the day but then proceeded to nighttime. After 9 months of treatment, Patient Two exhibited better performance on executive function, memory, and inhibition testing.

Similarly, Naeser et al. (2014) demonstrated that 11 chronic mild TBI patients participating in an open-protocol pilot study showed improvements in cognitive performance (inhibition, inhibition switching, verbal learning, and memory) following 18 t-PBM treatments targeting the default mode network (DMN), salience network (SN), and central executive network (CEN). These changes in cognition were accompanied by subjective reports of improved sleep (Naeser et al., 2014; Naeser et al., 2016). Naeser et al. (2023) continued to study the effect of t-PBM in TBI using an open protocol study in four former football players with evidence of possible chronic traumatic encephalopathy. Two patients reported sleep difficulties, while all four reported cognitive and memory issues. Following 1 month of treatment administered three times per week targeting the DMN, SN, and CEN, participants demonstrated changes using different measurements across patients. Patient One demonstrated increased SN functional connectivity using magnetic resonance imaging at 1 week and 1 month post-treatment. However, this decreased at 3 months post-treatment and increased again at 4 months post-treatment. This pattern mimicked the pattern of cognitive and mood changes. Patient Two also demonstrated increased functional connectivity in the SN at 1 week and 4 months post-injury. Patient Four also showed increased SN functional connectivity at 1 week, 1 month, 5 months as compared to pre-treatment. These increases in functional connectivity showed within subject correlations with cognitive (e.g., Stroop Test and Continuous Performance Test) and mood assessments, but not over time. Using magnetic resonance spectroscopy, n-acetyl-aspartate (NAA), a marker of mitochondrial oxygen consumption, was measured in Patient Two. At 1 and 6 weeks of treatment, NAA levels were increased in the anterior cingulate cortex but then decreased again at 12 weeks. The increased levels of NAA in the anterior cingulate cortex were inversely correlated with lower post-traumatic stress disorder and pain ratings. Improved sleep, measured with the Pittsburgh Sleep Quality Index (PSQI), demonstrated consistent improvements at 1 month after treatment in all four patients. These changes were accompanied by improvements in depression, pain, and post-traumatic stress. In addition, improvements in cognition were seen across multiple domains, including verbal learning and memory. Improvements in both cognition and sleep are seen in these studies; however, no evidence was provided to relate these two phenomena. These findings hint toward potential mechanistic insights to be explored as parallel changes in brain activity and cognition are seen. The placebo effect is also unknown in small studies and case reports; however, these results support promising changes after t-PBM that require further investigation using controls.

Morries et al. (2015) conducted a retrospective case series examining the effects of t-PBM on patients with chronic mild-to-moderate TBI. All participants demonstrated cognitive improvement as well as resolution of self-report of primary and middle insomnia. Although cognitive tests were not conducted, it was through examination of the patients that cognition may have improved. Similar subjective reports of improved sleep and the reduction of insomnia symptoms were recorded by Carneiro et al. (2019) in an open-protocol exploration of t-PBM application targeting the whole head in 10 patients diagnosed with severe TBI. Following 18 treatments delivered over 6 weeks, participants demonstrated increased cerebral blood flow, measured by transcranial Doppler. Although pre-treatment and post-treatment blood flow were not statistically significant, the blood flow was elevated with pronounced significant effects in the left hemisphere shown through peak systolic velocity. It is important to note that due to the diffuse nature of TBIs and the heterogeneity amongst the population (e.g., time post-injury, mechanism of injury) individual participants reacted different physiologically. More specifically, those who had sustained an injury between 7 and 8 months prior to treatment showed blood flow increases, while an individual who sustained an injury 18 months prior showed reduced blood flow. This highlights the importance of targeted t-PBM and understanding the underlying injuries and/or metabolic states of the treatment areas. In addition, they showed self-reported reduction of insomnia symptoms measured by the Beck Depression Inventory and neuropsychological improvements demonstrated by improved reaction time and cognition (e.g., verbal learning, executive function, visuospatial construction) 1 week post-treatment. Interestingly, some of these improvements lasted 3 months after the last session, including the reduction of insomnia symptoms and verbal learning and memory.

Two case reports presented by Chao (2019) documented significant reduction (greater than or equal two a six-point change) of insomnia symptoms on the Insomnia Severity Index for two patients exhibiting cognitive symptoms of Gulf War Illness following a 12 weeks open-protocol t-PBM treatment course consisting of 24 treatments. Individuals with Guld War Illness are often defined by the chronic multisymptom illness criteria, which includes a fatigue domain (i.e., fatigue and feeling unwell after physical exercise or exertion). These symptoms improved after t-PBM application compared to baseline. In addition, mood and cognitive domains are included in the chronic multisymptom illness criteria assessed by Kansas Gulf War Military History and Health Questionnaire. In Participant Two, difficulty concentrating and difficulty remembering recent information improved after treatment; however, they did not improve in Participant One. In addition, part of the neurological/cognitive/mood domains include problems getting to sleep or staying asleep, which improved in both participants.

Similarly, Martin et al. (2021) conducted a randomized, sham-controlled, double blinded study of t-PBM for 48 veterans exhibiting cognitive symptoms of Gulf War Illness. Three participants in the active treatment group expressed subjective experience of improved sleep, which were not noted in the sham group. Overall, no changes in sleep were seen after t-PBM treatment. Although the measures of sleep were subjective, multiple measurements were used to assess fatigue and sleep (see Table 1). One measure of attention/executive function, Digit Span Backward, demonstrated a significant difference between the treatment and sham group at 1 week and 1 month post-treatment. Other cognitive improvements post-treatment in domains such as attention/executive function, verbal memory and learning, and psychomotor/visuospatial memory showed a trend toward significance, but this is likely owed to a large heterogeneity within the population. While literature directly examining t-PBM’s effect on sleep as a primary outcome measure is currently scarce, secondary data collected in populations with diverse forms of TBI provides insight into the promise of future application of t-PBM for the treatment of sleep disorders. While most studies detailed positive and promising findings, there is variability amongst degree of improvement and some participants did not improve in sleep and/or cognition. It is not only important to note the small sample sizes and heterogeneity in the populations studied, but also the varying treatment parameters (Table 1) and shortage of physiological measurements to better understand these changes or lack of changes.

### 3.2 Cognitive decline

Three studies investigated the effects of t-PBM on sleep and cognition in individuals with cognitive decline (Nizamutdinov et al., 2021; Saltmarche et al., 2017; Zhao et al., 2022; see Table 2 for study details). While all described studies used a mix of cognitive assessments, two used subjective sleep measures (Nizamutdinov et al., 2021; Saltmarche et al., 2017) and one used an objective sleep measure (Zhao et al., 2022). Only one study was a controlled trial (Zhao et al., 2022), while two were open trial (Nizamutdinov et al., 2021; Saltmarche et al., 2017).

Nizamutdinov et al. (2021) studied individuals with mild-to-moderate dementia who were divided into t-PBM and sham groups for 8 weeks alongside comprehensive neuropsychological testing batteries. After at-home t-PBM treatment, participants demonstrated increased performance on multiple cognitive assessments, including logical memory, auditory verbal learning, executive functioning, and semantic memory (see Table 2). While this pattern of mild-to-moderate improvement did appear in several other cognitive assessments [e.g., digit symbol substitution, word fluency, logical memory test two delayed recall, and Mini Mental State Exam (MMSE)] after t-PBM, these findings were only trending toward significance and only some patients were described in detail as having positive changes in cognition. The sham group did not show statistically significant positive improvement overall or showed decline in cognitive assessments. In addition to these cognitive changes, the participants reported improvements in their sleep, which was reported after 1 week, including increased duration and mood reported by sleep duration and caregiver logs. Some descriptions included a more peaceful sleep and total sleep time increased by about 1 h. Some patients reported the cease of recurring nightmares. In addition, some patients did report feeling more wakeful during the day. These reports were not seen in the sham group. Although this is subjectively measured and descriptively recorded, it does merit that there was a difference in the reports between groups.

Similarly, Saltmarche et al. (2017) included participants who were diagnosed with dementia or AD and underwent 12 weeks of t-PBM over the DMN plus i-PBM. These participants showed improvement in cognition using two measures of objective cognitive impairment and sleep through self- and caregiver reports after the 12 weeks of treatment. After completion of treatment, five participants underwent a 4 week “no treatment” period, in which four participants did decline cognitively again as reported by family or caregivers and from cognitive assessments (i.e., Alzheimer’s disease Assessment Scale - Cognitive and MMSE; see Table 2 for more details). Although the sample size was small, these results provide invaluable data providing a basis for cognitive change post-treatment and for future studies.

More recently, a double-blind sham-controlled t-PBM study conducted by Zhao et al. (2022) showed that t-PBM applied to the right prefrontal cortex of individuals with subjective cognitive decline improves working memory and an indirect measure of sleep efficiency on the fifth day post-treatment. While no differences were seen in sleep efficiency, deep sleep and REM stage percentage, and wake-up time between the active and sham groups during the first 4 days of treatment, sleep efficiency (indirect measures, e.g., deep sleep and REM stage percentage) did improve within the active treatment group on the fifth day. This study is one of few to use objective measures of sleep (i.e., Sleepart, a wearable device that tracks sleep conditions). We caution that the sleep measures reported in this study used a device that has not been validated against the gold-standard in-lab polysomnography to our knowledge. The active treatment group also performed better than the control group in the accuracy and reaction time on the n-back test after treatment. Future studies should include sleep physiology measures to better understand the effect of t-PBM on sleep. Overall, the literature supports improvements in cognitive decline across subjects with dementia post-t-PBM (see Table 2), and ongoing clinical trials are aiming to address the clinical efficacy of t-PBM for this population (Iosifescu et al., 2023).

### 3.3 Other populations

Sleep outcomes were measured after t-PBM treatment in seven studies looking at anxiety (Maiello et al., 2019), depression (Hao et al., 2024; Guu et al., 2025), children with autism spectrum disorder (ASD; Pallanti et al., 2022), bipolar disorder (Mannu et al., 2019), Parkinson’s disease (Liebert et al., 2024), and healthy athletes (Zhao et al., 2012; see Table 3 for study details). Of the seven studies, five used PSQI as a measure of sleep (Zhao et al., 2012; Pallanti et al., 2022; Maiello et al., 2019; Hao et al., 2024; Guu et al., 2025), one used the Parkinson’s Disease Sleep Scale (Liebert et al., 2024), and one used subjective sleep measures (Mannu et al., 2019). Two studies were randomized and sham-controlled (Zhao et al., 2012; Guu et al., 2025), while the other five studies were open-label (Maiello et al., 2019; Mannu et al., 2019; Pallanti et al., 2022; Liebert et al., 2024; Hao et al., 2024).

Pallanti et al. (2022) explored sleep outcomes in 21 children with ASD. Sleep quality was measured using the PSQI; parents completed the PSQI at baseline and repeated the measure after 3 and 6 months. PSQI scores showed statistically significant improvement in sleep between baseline and at 3 months of treatment and after 6 months of treatment; however, no significant change was observed between 3 and 6 months. The treatment duration was 20 min twice a day for 5 days a week for 6 months, and children were encouraged to be involved in stimulating activities, such as playing games, drawing, or doing homework. At 3 months during treatment and after 6 months of treatment, children with ASD showed better performances on the Montefiore Einstein Rigidity Scale–Revised scale, which is a measure of cognitive and behavioral rigidity; however, this change remained stable between 3 and 6 months. With respect to another Scala per i Disturbi di Attenzione/Iperattività per Genitori, which measures inattention and hyperactivity/impulsivity, the children also showed better scores related to attention from baseline to 3 months and even more improvement after 6 months of treatment.

Maiello et al. (2019) explored sleep outcomes in 15 individuals with generalized anxiety disorder in an open-label study. Following 8 weeks of once daily t-PBM treatment, 11 participants reported improvement in anxiety symptoms and sleep quality, as shown by a significant decrease in sleep latency score on the PSQI. This result suggests a shorter amount of time to fall asleep after 8 weeks of t-PBM. While t-PBM is well-tolerated and little-to-no side effects are generally reported, two participants from this study did note side effects that included insomnia and one reported hypersomnia. However, these events were rated mild-to-moderate. While these findings are similar to previously described findings in other populations (e.g., Saltmarche et al., 2017; Morries et al., 2015), three participants reported side effects that had a negative effect on sleep.

In a randomized, sham-controlled study of 21 healthy female athletes, Zhao et al. (2012) noted improvements in global PSQI scores, and sleep quality as measured by subsets of the global PSQI after whole body photobiomodulation including t-PBM treatment 30 min every night for 2 weeks, compared to sham. Analysis of the subsets of the PSQI showed increases in subjective sleep quality, sleep duration, and decreased sleep latency in the treatment group. Sleep latency was also decreased in individuals with generalized anxiety disorder and may serve as an important marker of change in future studies. This study also provided data on a physiological change post-treatment by examining serum melatonin levels. Participants in the treatment group demonstrated improvement of serum melatonin levels compared to the sham group. Further, the improvement in serum melatonin levels was correlated with improvement in global PSQI score in the treatment group. While there were no cognitive measurements included, endurance performance was evaluated and showed some improvements in treatment compared to sham. This is one of the few studies to include a physiological marker of change in relation to sleep, but whole body photobiomodulation was used rather than t-PBM alone. This difference in the whole body photobiomodulation mechanism compared to t-PBM only may yield different results, but this area requires more research as there is very limited data comparing the two mechanisms (Bowen and Arany, 2023).

Two recent studies have looked at the effect of t-PBM in individuals with depression. Hao et al. (2024) conducted a pilot study where participants with major depressive disorder (MDD) received 2 weeks of open protocol treatment. In all 11 participants, Hamilton Depression and Anxiety Rating Scale scores in addition to sleep scores measured by the PSQI decreased after treatment. This pattern was also seen in the five participants who did complete the follow-up; in other words, these scores remained decreased at the 8 week post-treatment follow-up. However, PSQI scores remained decreased at both timepoints for those five participants but was not statistically significant compared to baseline. One participant experienced mild fatigue as a side effect, which does not support the general increases seen in previous literature yet is consistent with some mild-to-moderate sleep disruptions noted in the study by Maiello et al. (2019). Interestingly, five healthy controls without t-PBM treatment and five MDD participants before and after t-BPM underwent transcranial magnetic stimulation combined electroencephalography (TMS-EEG) over the left frontal pole to explore brain network connectivity. TMS-EEG data showed distinct brain network patterns, with controls exhibiting early connectivity in the left frontal pole and frontal-central cortex, and later involvement of the left posterior temporal and posterior regions. Pre-treatment, MDD patients showed altered patterns with reduced frontal-central outflow, a left frontal pole hub, and an additional right frontal pole hub. After treatment, their connectivity became more similar to controls, with earlier engagement of key regions. While left frontal pole outflow remained reduced, it gradually recovered toward the left central and occipital areas, and abnormal right frontal pole outflow disappeared.

The other recent study that explored the effect of t-PBM in individuals with depression was conducted by Guu et al. (2025). 48 patients with MDD were included in this randomized, double blind, controlled trial. Hamilton Depression Rating Scale, Beck Depression Inventory (with sleep sub-questions), and PSQI were assessed at baseline until week 12 after 8 weeks of treatment. The depression scores decreased in both the sham and treatment group, but a significant reduction in PSQI was noted only in the treatment group from week 2 onward compared to baseline. This significant reduction in the treatment group compared to sham lasted until week 12. While mild and transient adverse effects were noted for five patients (i.e., headache, tinnitus), none were related to sleep or wakefulness. While the low dosimetry or length of treatment may contribute to the lack of change in depressive symptoms seen in this study, changes in subjective sleep quality may serve as a more prominent or earlier effect post-treatment.

Liebert et al. (2024) reported that after 5 years of weekly t-PBM treatments, four out of six participants with Parkinson’s disease noted improvements in sleep quality. Two participants reported significant improvements in sleep quality, as shown by an increase of more than 20 points in the Parkinson’s Disease Sleep Scale. Additionally, all participants showed improvements in cognition, as shown by an increase in the Montreal Cognitive Assessment (MoCA) score. Specifically, all participants had an increased MoCA score after 1 year of treatment and a higher score at 5 years compared to baseline. In another study, Mannu et al. (2019) reported improvements in sleep and impulsivity through self-reports after t-PBM treatment in all four patients with type-I bipolar disorder who had been on lithium for at least 4 years and still experienced residual symptoms. The t-PBM treatment protocol was 20 min, twice a week for 4 weeks. Although no cognitive measures were administered, other clinically relevant self-reported improvements were seen including irritability, anxiety, and reduced anhedonia. In addition, lithium levels increased after 4 weeks of t-PBM, and so their psychiatrist lowered their respective doses. Baseline lithium levels were seen after reduction in dosage. The beneficial effects of t-PBM on the residual symptoms persisted after re-establishing baseline lithium levels, which hints that the improvements seen were unrelated to increased lithium levels. However, this relationship remains speculative and future research should consider including clinically relevant markers and physiological measures before and after t-PBM administration.

## 4 Discussion

Studies exploring the effects of t-PBM on individuals with TBI, cognitive decline, and other populations suggest both cognitive improvements and enhanced sleep quality. However, more research and the inclusion of individuals with sleep disorders (e.g., obstructive sleep apnea and/or insomnia) are critically needed. While direct studies on t-PBM’s effect on sleep are limited and there is some physiological evidence in the current literature, secondary outcomes from research in disorders comorbid with sleep and wakefulness difficulties suggest promising applications for treating sleep disorders. In addition, while the potential neural mechanism of t-PBM for increasing both sleep and wakefulness are encouraging but somewhat speculative, such physiological underpinnings require further exploration.

### 4.1 Summary of current literature

Across individuals with cognitive decline, TBI, healthy athletes, and other disorders (i.e., children with ASD and adults with anxiety, depression, bipolar disorder, and Parkinson’s disease), there is growing evidence that sleep and wakefulness (i.e., cognition, impulsivity, alertness, and memory) can generally be improved after t-PBM treatment. However, not all studies report positive findings. For example, two studies reported mild adverse events after t-PBM where sleep and/or wakefulness has been disrupted (Maiello et al., 2019; Hao et al., 2024). In addition, changes in sleep were not statistically significant in two studies (Martin et al., 2021; Hao et al., 2024). Two studies published as conference abstracts that were not excluded in this review also supported these findings. Bogdanova et al. (2017) found that the effect of transcranial plus intranasal red and near-infrared light intervention on cognition and sleep in veterans with chronic, mild traumatic brain injury, actigraphy measures of total sleep improved and sleep duration increased by 1 h (detailed by Naeser et al., 2016). In addition, cognitive performance improved as assessed by multiple neuropsychological tests measuring executive function and verbal memory. Liebel et al. (2022) found significant improvements in sleep quality were demonstrated through PSQI ratings in 49 former athletes with history of repetitive head injuries.

The current review focused on sleep outcomes and related cognitive processes only, and some studies reported more and/or primary outcomes, which may not have been reported if they were not related to the aim of the review. For example, Liebert et al. (2024) reported improvements in motor skills (i.e., mobility and balance) that were primary outcomes of the study. For individuals with Parkinson’s disease, improvements in motor skills were seen after 5 years of treatment, which is critical for this population. Four of five participants who completed long-term treatment reported improvements in both quality of life and sleep. Very few outcome measures deteriorated after 5 years of treatment and no side effects were noted. This study also highlights the importance of studying long-term maintenance of improvement with t-PBM in clinical populations, which is also critical to the field. The other important findings on motor skills, mood, and overall quality of life yield the need for large, long-term clinical trials. While there has been literature supporting cognitive changes after t-PBM (Lee et al., 2023; Salehpour et al., 2022), more literature is needed to understand the relationship of post-treatment changes in sleep and related cognition. However, the field of t-PBM for sleep faces important challenges that need to be addressed to develop t-PBM as a therapeutic tool for sleep. Additionally, in most studies identified in this review, sleep was typically a secondary measure and/or was assessed using caregiver daily logs or subjective measures (Naeser et al., 2011; Zhao et al., 2012; Naeser et al., 2014; Morries et al., 2015; Saltmarche et al., 2017; Mannu et al., 2019; Carneiro et al., 2019; Chao, 2019; Maiello et al., 2019; Martin et al., 2021; Nizamutdinov et al., 2021; Pallanti et al., 2022; Naeser et al., 2023; Hao et al., 2024; Liebert et al., 2024; Guu et al., 2025). Only one (Zhao et al., 2022) study included an objective sleep measure, using a wearable device that can measure sleep efficiency and the percentage of REM sleep. However, to our knowledge this device has not been validated against in-lab polysomnography.

In addition, scarce physiological changes have been recorded in addition to behavioral assessments collected. Three studies included a neuroimaging measure (Carneiro et al., 2019; Naeser et al., 2023; Hao et al., 2024) and two included other physiological measures (Mannu et al., 2019; Zhao et al., 2012). Furthermore, the neurobiological basis of how t-PBM can improve sleep is somewhat speculative. There is strong evidence supporting the effect of t-PBM on the brain, but more research is needed to determine how these underlying neurobiological mechanisms are related to sleep and wakefulness. Future studies should include more quantitative sleep assessments that span across sleep characteristics as well as physiological data, such as actigraphy or sleep electroencephalography. Although not all studies utilized objective measures of change in sleep quality and wakefulness, the results carry scientific merit and should be integrated into future studies, given the key role of disrupted sleep and excessive sleepiness in many clinical populations.

### 4.2 Transcranial photobiomodulation in animal models during sleep and wake

Literature on t-PBM in animal models during sleep and wakefulness is still in its infancy, and some of the mechanisms of action remain speculative. Two recent reviews (Moro et al., 2022; Valverde et al., 2023) have detailed the current state of the literature and outlined the growing evidence to support t-PBM in enhancing glymphatic clearance in animal models. However, this review does not report behavioral and cognitive outcomes related to these models. In addition, t-PBM has mostly been applied to animal models during wakeful states (e.g., Sipion et al., 2023) and so there is a need to understand the application of t-PBM during the night as suggested by Valverde et al. (2023). Mixed behavioral and cognitive results have been reported in animal models after t-PBM (e.g., animal models of aging and Alzheimer’s disease reviewed by Rodríguez-Fernández et al., 2024), but the results are leaning toward improvements in behavior and cognition. The mixed results are likely owed to unstandardized t-PBM treatment parameters and different animal models. The current animal model and human literature are growing in parallel and are promising. Preclinical animal research has great potential to explore t-PBM during sleep and wakeful states to inform on the neurobiological and behavioral mechanisms of t-PBM in humans. Future research should include cognitive and behavioral measures in animal models before and after t-PBM during sleep and wakefulness, to understand the relationship between these phenomena.

### 4.3 The future of transcranial photobiomodulation on sleep, wakefulness, and related cognition

Current research interests point toward the use of non-invasive neuromodulation strategies for sleep in both healthy and clinical populations (Cheng et al., 2022; Luff and De Lecea, 2024). t-PBM enhances cerebral mitochondrial function and is mechanistically very different than other neuromodulation techniques, such as transcranial magnetic stimulation, that directly alter neuronal activity. Therefore, t-PBM and other neuromodulation techniques have different consequences on sleep-wake cycles and memory. t-PBM has an excellent safety profile and is well-tolerated (Cassano et al., 2022b), with only some contraindications such as lesions directly under the site of irradiation and light-activated medications (e.g., criteria listed in Spera et al., 2021). While there have not been any reported severe side effects of long-term exposure, t-PBM does have a biphasic dose-response (Huang et al., 2009) so too high or low of a dose may lead to no response or an unwarranted response. There is some evidence that different t-PBM doses different effects on brain activity (Iosifescu et al., 2022), but still more research is needed to determine the optimal dose to avoid unwanted effects or lack thereof.

There is also growing evidence for the use of t-PBM for sleep; t-PBM is the only neuromodulation strategy that directly affects cerebral mitochondria, which has implications on blood flow, ATP production, and the brain’s glymphatic system (Hamblin, 2016; Salehpour et al., 2022). To be applied clinically, many unknowns need to be addressed, including the heterogeneity of treatment parameters across subjects and studies, treatment settings, and time-of-day administration. Within both human and animal studies, different wavelengths, radiation power, treatment design, application surface, and mode (i.e., continuous versus pulsed light) are used. This calls for the need to determine the optimal treatment parameters in the field, although there is some movement toward doing so (Weerasekera et al., 2024; Cassano et al., 2022a). Future studies should compare t-PBM parameters and treatment schedules at different times of day to improve treatment outcomes and should investigate whether t-PBM works synergistically with other interventions aimed at improving sleep and wakefulness. In addition to objective sleep measures, studies should include physiological outcomes, such as neuroimaging and polysomnography. However, studies must carefully consider t-PBM during sleep, which may be hindered due to safety concerns where the lasers or LEDs may irradiate into unwanted areas, such as the eyes. This may pose a safety concern. t-PBM using LEDs may serve as the most feasible option for applying t-PBM during sleep. Therefore, studies should focus first on applications of t-PBM during wake to measure both sustained wakefulness and sleep quality after applications at different times of day before exploring the feasibility of t-PBM during sleep.

### 4.4 Limitations of current systematic review

While cognition is often a primary outcome for t-PBM research across clinical and healthy populations (Hamblin, 2016; Lee et al., 2023; Salehpour et al., 2022), the direct effect of t-PBM on wakefulness and sleep and related cognition is relatively more recent, and the literature is scarce. Further, the understanding of basic mechanisms for the complex cascade of cellular processes in the context of wakefulness and sleep after t-PBM application parallels the evolving development of parameters for treatment protocols (see Tables 1–3 for details on study parameters); this highlights two limitations including lack of optimal treatment parameters and the speculative nature of the underlying mechanisms on t-PBM for sleep and wakefulness. Further, lack of standardization of t-PBM parameters, small number of sham-controlled designs, and diverse study populations add to the limitations of the current review.

The selection of literature included in the review may lead to bias since sleep is typically a secondary measure and can be potentially overlooked. However, we believe we have included the relevant literature and have reported all results whether they were secondary or primary and positive or negative. Some results may be inconclusive due to the small sample sizes, self-report measures, and/or different treatment parameters. In addition, animal models were not included in this review; however, recent reviews (Moro et al., 2022; Valverde et al., 2023) have detailed the state of the current literature, which is still growing and in its early stages. And so, the purpose of this current review is exploratory and descriptive in nature, which allows us to explore (1) the emerging and broad evidence of t-PBM’s effect on sleep and wakefulness and related cognition with great detail, (2) the potential mechanisms underlying these novel changes, and (3) the gaps in the literature which future studies can benefit from and serve as a basis for future systematic reviews.

Most of the studies reviewed have correlation designs and some have control conditions (see Tables 1–3) and four were randomized controlled trials (Zhao et al., 2022; Martin et al., 2021; Nizamutdinov et al., 2021; Guu et al., 2025); however, more large sample randomized controlled trials are needed including individuals with sleep disorders. Future studies should consider objective measures of sleep, sleep disturbance, and changes to sleep stage or sleep physiology. Future studies should also consider time of day or night of t-PBM application and the optimal parameters for improvements for sleep and/or wakefulness, as the current literature is still in its infancy. Although it is difficult to disentangle sleep disorders and other psychiatric conditions, it is important to also explore the changes in individuals with primary sleep disorders, such as obstructive sleep apnea, where sleep is likely the primary complaint.

## 5 Conclusion

Transcranial photobiomodulation shows promise as a method for improving sleep, wakefulness, and associated cognition. Overall, t-PBM may offer a promising, non-invasive method to enhance wakefulness by improving brain health, optimizing cerebral metabolism, and supporting cognitive function and memory consolidation, making it particularly useful for individuals experiencing chronic fatigue, cognitive impairment, or sleep disorders. More specifically, impaired sleep has been seen as a risk factor for cognitive decline and memory impairments, making the need to understand t-PBM’s effect on individuals with sleep impairments of clinical relevance to both healthy and diseased populations. However, more robust and larger-scale clinical studies are needed to fully establish its efficacy and optimal protocols for different clinical populations, including those with sleep disorders, as the current literature is still in its infancy. In addition, some data has shown side effects of t-PBM where sleep and/or wakefulness has been disrupted (Maiello et al., 2019; Hao et al., 2024). And so, while the current literature supports a general improvement in sleep, wakefulness, and related cognition, more research is needed to determine the effect of t-PBM on sleep and wakefulness. Future research should incorporate objective sleep measures, time of day considerations, testing of optimal treatment parameters, and physiological outcomes after t-PBM in sleep disorders.
