# Supplementary material for: Enhancing sleep, wakefulness, and cognition with transcranial photobiomodulation: a systematic review
Source: Front Behav Neurosci. 2025 Jul 31;19:1542462. doi: 10.3389/fnbeh.2025.1542462 (PMC12350269; doi:10.3389/fnbeh.2025.1542462)
Supplement: Supplementary file 1 [file Supplementary_file_1.docx]

| Author(s) | Year of Publication | Country of Origin | Aims | Study population | Sample Size (if applicable) | Methods | Intervention Type/Duration | Outcomes | How Outcomes are Measured |
| --- | --- | --- | --- | --- | --- | --- | --- | --- | --- |
| Naeser MA, Saltmarche A, Krengel MH, Hamblin MR, Knight JA | 2011 | USA | Improve cognition in chronic TBI patients using transcranial light stimulation | Chronic TBI Patients | 2 | Administration of red/NIR LED light to midline sagittal areas, 3 square cluster shapes applied with the device with the following specifics for device one “40 NIR 870 nm diodes, 12.25 mW each, and 9 red 633 nm diodes, 1 mW each” or the second device circular shaped area application (1- 3 areas) and specifics “52 NIR 870 nm diodes, and 9 red 633 nm diodes, 12–15 mW each.” | Treatment duration varied. Patients received this treatment over multiple visits and have been on treatment now over a series of years, however, improvement was noted in patient one as early as 3 days of use. Patient one cannot stop using the device nightly for more than 2 weeks or she will regress. | Patient 1 increased her sustained attention and improved sleep Patient 2 showed improvement after treatment in executive functioning, inhibition, inhibition accuracy | Patient 1: measured sustained attention by increased time spent on computer, improvement in sleep is measured through self report Patient 2 showed improvement through the cognitive task "Stroop" |
| Naeser MA, Zafonte R, Krengel MH, Martin PI, Frazier J, Hamblin MR, Knight JA, Meehan WP 3rd, Baker EH. | 2014 | USA | Improve cognition using scalp application of red and near-infrared (NIR) light-emitting diodes (LED) in chronic mild TBI patients | Chronic Mild TBI patients | 11 | Each LED cluster head (5.35 cm diameter, 500 mW, 22.2 mW/cm2) was applied for 10 min to each of 11 scalp placements (13 J/cm2). LEDs were placed on the midline from front-to-back; and bilaterally on frontal, parietal, and temporal areas. Neuropsychological testing was performed pre-treatment, and at 1 week, and 1 and 2 months after the 18th treatment. | Treatment administered for 3 times a week for 6 weeks with 18 sessions | Improvements in cognitive performance (inhibition, inhibition switching, verbal learning, and memory) as well as a self reported sleep improvement as well as fewer PTSD symptoms | Sleep was a self report metric and cognitive testing was administered through the following tests Stroop Test, California Verbal Learning Test-II, Delis–Kaplan Executive Function, Controlled Oral Word Association Test, Digit Span, Forwards and Backwards, Wechsler Adult Intelligence Scale. Improvements reported seen on Stroop and CVLT-II. |
| Naeser M. A., Martin P. I., Ho M. D., Krengel M. H. , Bogdanova Y., Knight J. A., Hamblin M. R., Fedoruk A. E. , Poole L. G. , Cheng C. H., and Koo B. B. | 2023 | USA | Examine cognition, behavior/mood changes Post-t-PBM and look at MRI resting state connectivity | Traumatic Encephalopathy Patients | 4 | Four ex-football players with traumatic encephalopathy syndrome/possible chronic traumatic encephalopathy received red/near-infrared t-PBM to scalp 3 times a week for 6 weeks | Treatment administered 3 times a week for 6 weeks | There was significant improvement at one month on PTSD, depression, pain and sleep scores. Increase in SN functional connectivity after 1-month treatment , additionally Improvements in cognition were seen across multiple domains. | Cognition was measured using Color-Word Interference (Stroop), California Verbal Learning Test-II, Trail-Making Test, Controlled Oral Word Association Test, Connors’ Continuous Performance Test, Brief Visuospatial Memory Test- Revised. Sleep was measured through the Pittsburgh sleep quality index. Depression was measured through the beck depression inventory- II. Additional metrics is the short form McGill pain questionnaire and PTSD checklist civilian. |
| Morries, L. D., Cassano, P., & Henderson, T. A. | 2015 | USA | Review treatment of TBI with use of a high power NIR laser | Chronic mild to moderate TBI | 10 | Treatments took place over the course of 2 months using a high-power NIR laser "(13.2 W/0.89 cm(2) at 810 nm or 9 W/0.89 cm(2) at 810 nm and 980 nm)" | the administration for the study treatment was 8-12 min., 10 visits (6/10 participants) or 20 visits (4/10 participants) | Improvements were seen in the symptoms of headache, sleep disturbance, cognition, mood dysregulation, anxiety, and irritability. | Quick Inventory of Depressive Symptomatology Self-Report (QIDS-SR) was administered to all participants and BDI administered to 7/10 participants. |
| Carneiro AMC, Poiani GC, Zaninnoto AL, Osorio R. L., Oliveira ML, Paiva WS, Zângaro RA. | 2019 | Brazil | Evaluate hemodynamic conditions and neurocognitive changes before and after t-PBM treatment | Severe TBI | 10 | Photobiomodulation therapy was administered. Neuropsychological testing was administered pre, post, and late photobiomodulation therapy. Doppler was used to measure CBF. Pre and one week post t-PBM | Treatment administered 3 times a week for 6 weeks | Found an alteration in the cerebral blood flow (CBF) as well as a consequent increase of the cerebral oxygenation that helped to improve the cerebral function. Improvement trends seen in some aspects of cognitive function. | Doppler used at 7.5 MHz in B-mode with linear probe. Beck anxiety inventory, beck depression inventory were administered questionnaires. Neuropsychological tests administered include, Stroop, Trail making A and B, digit symbol, RAVLT, complex Rey figure, verbal fluency. |
| Chao LL. | 2019 | USA | To describe improvements in GWI symptoms in Gulf War Veterans after photobiomodulation | Gulf War Illness Veterans | 2 | Patients had thorough clinical interviews and questionnaires about PTSD, Pain, Insomnia, Military History and Health, Alcohol and Substance abuse, TBI, and MDD. Patients were instructed to use the | Treatment administered every other day for 12 weeks | After 12 weeks of PBM treatments, Participant 1 no longer met the criteria to be considered a Kansas Gulf War Illness case. Although this participant remained a mild-moderate case at Week 12, his mood-cognitive domain score, pain, sleep, and fatigue symptoms all improved  Participant 2 was not considered at baseline a Gulf War Illness case but was a severe chronic multisymptom case. After 12 weeks of t-PBM, the participant was re-classified as a mild-moderate case because he rated most symptoms as “mild” or “moderate.” At Week 12, his fatigue, pain, and mood-cognitive, pain and sleep symptoms all improved. | GWI severity index, Kansas Gulf War Military History and Health, Brief Pain Inventory and Insomnia Severity Index |
| Martin Paula I. , Chao Linda , Krengel Maxine H. , Ho Michael D. , Yee Megan , Lew Robert , Knight Jeffrey , Hamblin Michael R. , Naeser Margaret A. | 2021 | USA | Investigated whether photobiomodulation applied transcranially could improve cognition, and health symptoms in Gulf War Illness | Gulf War Illness Veterans | 48 | This study is a blinded, randomized, sham-controlled trial using Sham or Real, red/NIR light-emitting diodes (LED) applied transcranially. Administration of neuropsychological testing and psychosocial questionnaires. | 15 half hour treatments twice a week for roughly 7.5 weeks | Improvements on a multitude of cognitive outcomes for both the sham and real groups was reported 1 week after the 15^th^ treatment however after 1 month improvements were only noted in the real treatment group and sham regressed. Similarly at one month those with a PCL-C PTSD score over 36 at baseline, sham and treated showed reduction in PTSD scores at 1 week but then at 1 month only those on active treatment showed continued improvement and reduction in PTSD, sham regressed. | Digit Span, Delis-Kaplan executive function test trails, Color-Word interference (Stroop), California verbal learning test II, Connor's continuous performance test II, Rey osterrieth complex figure test  PCL-C PTSD checklist civilian, Visual analog pain rating scale (VAS), Short form McGill pain questionnaire, West Haven-Yale Multidimensional Pain Inventory (WHYMPI), Multi-dimensional fatigue inventory, Beck Depression Inventory II (BDI-II), Health Symptom Checklist (HSC), Veterans RAND 36 item health survey, SF-36V Plus, Pittsburgh Sleep Quality Index (PSQI), Epworth Sleepiness Scale, Karolinska Sleepiness Scale |
| Nizamutdinov, D., Qi, X., Berman, M. H., Dougal, G., Dayawansa, S., Wu, E., Yi, S. S., Stevens, A. B., & Huang, J. H. | 2021 | USA | Examine the safety and treatment benefits of transcranial NIR treatment for dementia | Early and moderate dementia | 60 | Neuropsychological testing was done before and after treatment. Active treatment consisted of a low power NIR light simulation with active photo biomodulation twice daily for 6 minutes for 8 weeks. Sham was conducted in the same manner. | Twice a day daily for 6 minutes for 8 weeks | Treatment with active device resulted in improvements of cognitive outcomes shown in neuropsychological testing. Trending toward significant improvements in the active treatment arm included, CCT, LMT-II, DS Forward and Backward, Digit symbol substitution, word fluency. Significant improvements in cognition in the active treatment arm included Auditory verbal learning test, Boston naming test, TMT- A & B, and LMT-I.  Caregivers to the patients also reported less anxiety, improved mood, more energy and positive daily routine after 2 to 3 weeks of treatment. | Administered the MMSE, Logical Memory tests immediate and delayed recall, auditory verbal Learning tests, Boston naming test, trail making A and B, Clock Copying test, digit span forward and backward, Wais-R digit symbol substitution, word fluency |
| Saltmarche, A. E., Naeser, M. A., Ho, K. F., Hamblin, M. R., & Lim, L. | 2017 | Canada | Assess whether patients with mild to moderate dementia would benefit from NIR photobiomodulation | Mild to moderate dementia or AD | 5 | Treatment was conducted with light-emitting diode devices combining transcranial and intranasal photo biomodulation in patients with mild to moderately severe cognitive impairment | 2 times a week for first 2 weeks, 1 time a week for last 10 weeks (transcranial); 1 time a day for 12 weeks (intranasal), as well as a follow-up no-treatment, 4-week period. | There was significant improvement in cognition after 12 weeks. Additionally, “Increased function, better sleep, fewer angry outbursts, less anxiety, and wandering were reported post-PBM.” However noteworthy there was a decline after no-treatment follow up period. | Measured by MMSE and Alzheimer's Disease Assessment Scale (ADAS-cog) |
| Zhao X, Du W, Jiang J, Han Y. | 2022 | China | To test whether PBM therapy targeting the frontal cortex could improve sleep and cognitive function | Subjective Cognitive Decline | 58 | Participants were divided into real light therapy and sham treatment. The treatment was applied for 6 continuous days and the N-Back task was administered to assess working memory | Laser treatment for 12 min one time a day for six continuous days | The results showed increased performance in the real treatment group increased in accuracy and shorter reaction time. Not statistically significant between groups but there was improvement on the 5^th^ day seen for sleep efficacy and REM in the treatment group | Sleep monitoring with wearable device (Sleepart) and N-Back task for cognitive assessment |
| Zhao J, Tian Y, Nie J, Xu J, Liu D. | 2012 | China | Determine the effect of whole body red-light therapy on athletes sleep and endurance | Healthy athletes | 20 | Subjects randomly assigned to red light or non-red light treatment (placebo) while undergoing the 14 days of treatment he subjects also did 12 exercise sessions | Treatment is applied for 14 days, note that this treatment is administered to the whole body. | Improvements were seen in sleep, serum melatonin. A trend was seen (although not significant) toward improvement was seen in athlete endurance. Red light improved sleep quality and duration and the global PSQI. | PSQI was used to assess sleep metrics. Serum Melatonin was measured as well as using a 12 minute run. |
| Pallanti, S., Di Ponzio, M., Grassi, E., Vannini, G., & Cauli, G. | 2022 | Italy & USA, Switzerland | Examine if t-PBM is effective in reducing behavioral and cognitive rigidity in ASD patients | Children with Autism Spectrum Disorder | 21 | Administration of t-PBM 5 treatments a week for 6 months for 20 min at a time after 3 months and 6 months patients are given questionnaires on ASD and Sleep | Treatment 5 times a week for 6 months. | Patients showed improvement in ASD severity (in CARS) after t-PBM treatment and additionally a reduction in behavioral and cognitive rigidity and an improvement in attentional functions and in sleep quality | The measurements used were Childhood Autism Rating Scale (CARS), the Home Situation Questionnaire-ASD (HSQ-ASD), the Autism Parenting Stress Index (APSI), the Montefiore Einstein Rigidity Scale−Revised (MERS−R), the Pittsburgh Sleep Quality Index (PSQI) and the SDAG, to evaluate attention |
| Maiello, M., Losiewicz, O. M., Bui, E., Spera, V., Hamblin, M. R., Marques, L., & Cassano, P. | 2019 | USA | Test the anxiolytic effect of t-PBM with NIR in subjects suffering from generalized anxiety disorder | Generalized anxiety disorder patients | 15 (12 completed the trial) | Patients self-administered t-PBM daily for around 20 minutes for 8 weeks with an LED cluster headband | Treatment once daily for eight weeks | There was improvement in anxiety seen through a reduction of scores on the SIGH-A and CGI-S and improvement in sleep. | Anxiety was assessed with the Hamilton Anxiety Scale (SIGH-A) and the Clinical Global Impressions-Severity (CGI-S) subscale and sleep was assessed with the Pittsburgh Sleep Quality Index (PSQI) subscale |
| Guu, T.-W., Cassano, P., Li, W.-J., Tseng, Y.-H., Ho, W.-Y., Lin, Y.-T., Lin, S.-Y., Chang, J. P.-C., Mischoulon, D., Su, K.-P. | 2025 | Taiwan, UK, USA | To assess the feasibility, safety, and effectiveness of a self-administered wearable t-PBM for MDD and sleep disturbances | Adults with major depressive disorder (MDD) | 48 (25 t-PBM, 23 sham) | Randomized, double-blind, sham-controlled clinical trial conducted across two hospitals in Taiwan | LED t-PBM (850 nm) via wearable headband, 20–40 minutes daily for 8 weeks | Significant improvement in sleep quality (PSQI) in the t-PBM group from week 2 onward; no significant difference in depression symptoms vs. sham | PSQI, HAM-D, BDI at baseline, weeks 2, 4, 8, and 12 |
| Hao, W., Dai, X., Wei, M., Li, S., Peng, M., Xue, Q., Lin, H., Wang, H., Song, P., Wang, Y. | 2024 | China | To investigate the effects of t-PBM on clinical symptoms and time-varying EEG network connectivity in MDD patients | Adults with MDD, treatment-resistant or drug-naïve | 11 (5 completed full follow-up) | Pilot study using TMS-EEG with pre- and post-intervention neuropsychological testing | LED t-PBM (820 nm) targeting the left frontal pole (Fp1); 16 minutes daily for 14 consecutive days | Significant improvement in depressive and anxiety symptoms; normalization of brain network activity; slight but not statistically significant improvement in sleep | HAMD, HAMA, PSQI (baseline, post-treatment, 4-week, and 8-week follow-up); TMS-EEG before and after treatment |
| Mannu, P., Saccaro, L. F., Spera, V., and Cassano, P. | 2019 | USA | Assess the treatment of t-PBM on patients with bipolar disorder | Bipolar disorder, type I, patients | 4 | Treatment using NIR t-PBM 20 minutes twice a week for four weeks. | Treatment is conducted 2 times a week for 4 weeks | All patients reported a reduction of anhedonia/apathy and increase of libido, together with isolated improvements in anxiety, sleep, irritability, and impulsivity.  Lithium levels went up after four weeks of treatment compared to baseline, the psychiatrist then reduced the lithium medication that the 4 patients were on, and then the lithium levels were comparable to baseline. | Residual symptoms and lithium serum levels |
| Liebert, A., Bicknell, B., Laakso, E.-L., Tilley, S., Heller, G., Kiat, H., Herkes G. | 2024 | Australia | To assess long-term effectiveness of photo biomodulation therapy after five years of continuing therapy. | Parkinson’s disease patients | 7  (6 used in analysis) | Home based self-applied photobiomodulation therapy | Patients use the treatment three times per week for five years. Patients, however, had varying levels of consistency. | Sleep quality was slightly improved in four out of six participants and all participants had a higher MoCA scores at 5 years than at baseline, Most motor outcome measures were improved at five years compared to baseline, with walk speed, stride length and the step tests significantly improved. | Cognition was measured with the MOCA, sleep quality was measured with the Parkinson's disease sleep scale (PDSS) Mobility was measured for range of clinical motor skills including the assessment MDS-UPDRS-III |
