# Supplementary figures and images for: Enhancing sleep, wakefulness, and cognition with transcranial photobiomodulation: a systematic review
Source: Front Behav Neurosci. 2025 Jul 31;19:1542462. doi: 10.3389/fnbeh.2025.1542462 (PMC12350269; doi:10.3389/fnbeh.2025.1542462)

**Supplemental Figure 1.** PRISMA flow-chart of the article selection process.

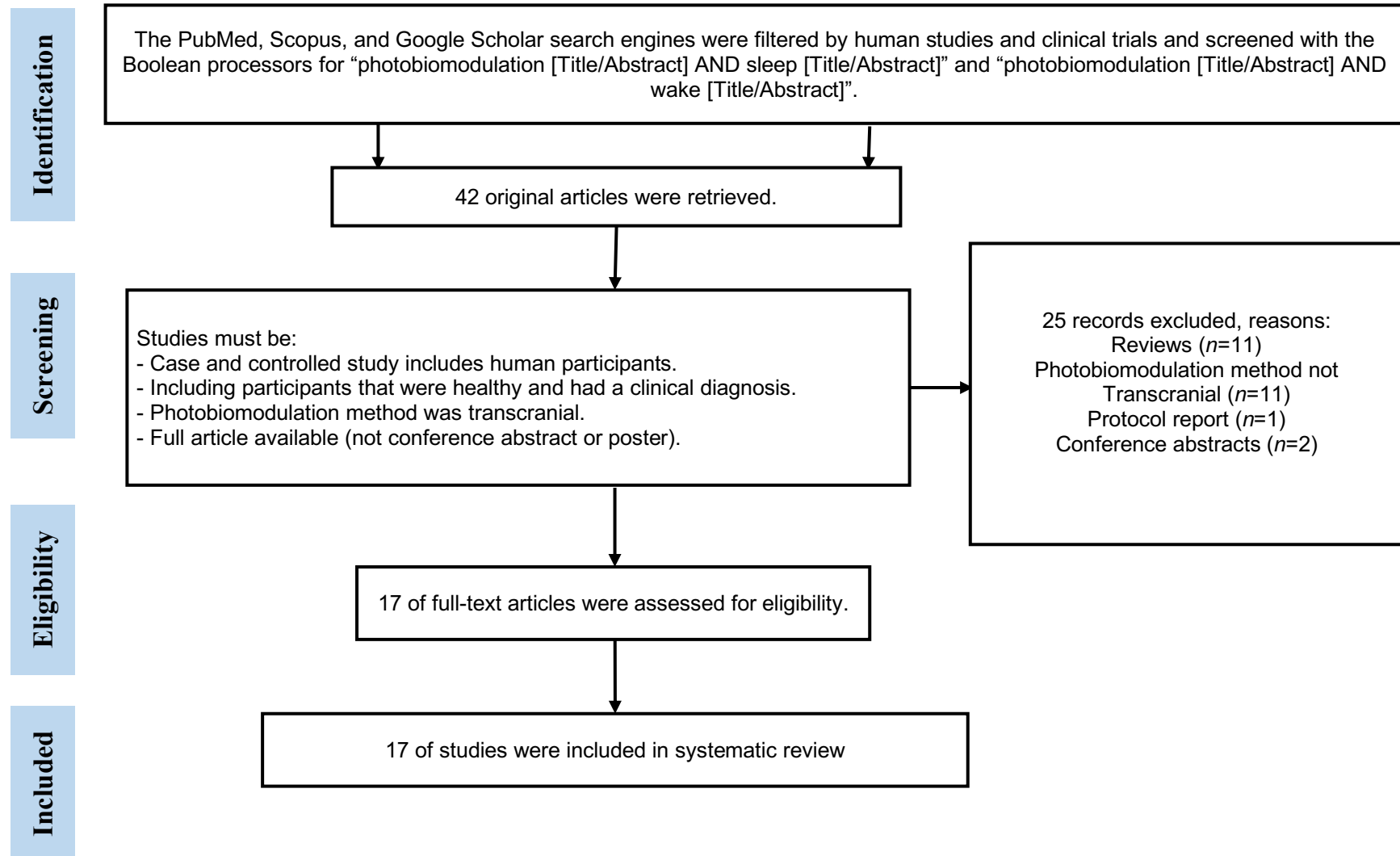

Supplement: Supplementary file 2 [file Image_1.pdf]
